# Efficient saccade enhances performance in clinical dynamic visual acuity test

**DOI:** 10.3389/fnins.2026.1834160

**Published:** 2026-08-07

**Authors:** Ruoyu Li, Zesong Wang, Yuexin Wang

**Affiliations:** 1 Department of Ophthalmology, Peking University Third Hospital, Beijing, China; 2 Peking University Health Science Center, Beijing, China

**Keywords:** dynamic visual acuity, eye movement, functional vision, saccade, smooth pursuit

## Abstract

**Purpose:**

To investigate the eye movement pattern across different speeds during clinical dynamic visual acuity (DVA) tests and their relationship with dynamic visual performance.

**Methods:**

Twenty young adults with normal or corrected-to-normal visual acuity underwent clinical DVA tests at 20, 40, 60, and 80 degrees per second (dps). Eye movement was recorded using a table-mounted eye tracker. Smooth pursuit- and saccade-related parameters were calculated from the gaze trace, and the relationships between eye movement parameters and dynamic visual performance were analyzed.

**Results:**

DVA was significantly better at 20 dps than at higher speeds (*p* < 0.05 for all comparisons). Eye movement patterns and parameters varied with target velocity, as smooth pursuit dominated at low speeds and saccades became more prominent at high speeds. At 20 dps, DVA correlated negatively with saccade displacement (*r* = −0.523, *p* = 0.022) and displacement per saccade (*r* = −0.528, *p* = 0.020). During the DVA test at 80 dps, positive correlations emerged between DVA and saccade period (*r* = 0.521, *p* = 0.022), duration (*r* = 0.500, *p* = 0.029), and displacement (*r* = 0.483, *p* = 0.036). Increased saccade episode (*β* = 0.540, *p* < 0.001) and time (*β* = 27,333, *p* < 0.001) were associated with greater odds of identification error at 20 dps. At 80 dps, higher smooth pursuit velocity was associated with increased odds of identification error (*β* = 0.030, *p* = 0.016).

**Conclusion:**

Eye movement strategies adapt dynamically to target speed. Quick and efficient saccades support improved dynamic visual performance, although high-amplitude saccades and pursuit velocity disrupted tracking at high speeds. These findings offer insights for optimizing clinical DVA tests and enhancing the clinical utility of DVA as a functional vision indicator.

## 1 Introduction

Dynamic visual acuity (DVA) describes the ability to discern fine details in a visual target during relative motion between the observer and the target. This capacity is crucial for daily activities like driving, walking, and sports, which require the real-time perception of and response to moving objects (Land, 2006; Ishigaki and Miyao, 1993; Shinar and Schieber, 1991). Consequently, DVA may better reflect functional vision in real-world conditions and prove more sensitive to visual impairment than static visual acuity (Ao et al., 2014; Wang et al., 2023a). With these advantages, DVA has emerged as a valuable indicator of functional vision in clinical contexts such as myopia, refractive surgery, cataract, and dry eye (Wang et al., 2022, 2023b; Ren et al., 2023).

DVA can be assessed with dynamic objects during head fixation (dynamic-object DVA) or static objects during head rotation (static-object DVA) (Chen et al., 2022). These paradigms engage different oculomotor systems, pursuit versus vestibulo-ocular reflex, and may measure distinct aspect of dynamic vision. Dynamic-object DVA is primarily used in clinical ophthalmology. In the test, patients are required to identify the orientation of a moving letter, typically an E or a Landolt C. DVA represents the smallest optotype size that can be accurately recognized. During testing, the head remains stationary while the eyes are free to track the moving optotypes. Eye movements are critical for dynamic vision, with saccades and smooth pursuit being particularly important (Land and McLeod, 2000; Goettker and Gegenfurtner, 2021; Shanidze and Verghese, 2024). Saccades are rapid, brief shifts of gaze toward a new location, whereas smooth pursuits are slower, continuous motions that keep moving targets near the fovea (Spering, 2022; de Brouwer et al., 2002).

Previous research indicates that oculomotor patterns strongly influence DVA. Early work by Ludvigh and Miller (1958). established that visual acuity decreases during ocular pursuit of a moving target, while Brown (1972a, 1972b) demonstrated that DVA and eye movement patterns are influenced by target contrast and peripheral visual processing. Uchida et al. (2013) found that athletes exhibit superior DVA than non-athletes, which they attributed to enhanced tracking ability. Palidis et al. (2017) further demonstrated that specific pursuit and saccade patterns correlate with better DVA performance. However, these findings were obtained under laboratory conditions using DVA test procedures and outcome calculation methods that differ substantially from those employed in clinical practice (Wang et al., 2022). The eye movement patterns occurring during clinically administered DVA tests have not yet been characterized.

This study aims to examine how eye movement patterns vary with target speed during the clinical DVA test and investigate their relationship with moving optotype identification. We defined a comprehensive set of parameters to quantify smooth pursuit and saccades, then evaluated their contributions to overall DVA performance and trial-by-trial identification accuracy. By linking specific eye movement features to dynamic visual performance, this work establishes a foundation for optimizing the clinical DVA test and its interpretation.

## 2 Methods

### 2.1 Participants

Adult participants aged 18 to 35 years with normal or corrected-to-normal visual acuity were enrolled. Exclusion criteria comprised: (1) monocular corrected distance visual acuity (CDVA) worse than 0 logMAR in either eye; (2) severe refractive errors, defined as a noncycloplegic subjective refraction sphere greater than +3.00 diopter (D) or less than −6.00 D, or astigmatism exceeding 3.00 D; (3) a history or presence of severe ocular diseases such as glaucoma, corneal abnormalities, retinal diseases, or macular degeneration; (4) systemic conditions known to impair visual function; (5) cognitive or vestibular impairments that could affect task performance; and (6) an inability to follow examiner instructions or to visually track dynamic stimuli. All procedures adhered to the Declaration of Helsinki and received approval from the Institutional Review Board. Written informed consent was obtained from all participants before the study.

### 2.2 Dynamic visual acuity test

The DVA test used in this study measures dynamic-object DVA. Its stimuli, procedure, and result calculation formula matched those of earlier research that has been widely adopted in clinical ophthalmology (Wang et al., 2022). Visual targets were presented on a 24-inch in-plane switching monitor with a 120 Hz refresh rate. A custom MATLAB program (version 2017b, MathWorks, USA) incorporating the Psychtoolbox generated moving letter E optotypes, which adhered to the configuration and size specifications of a standard logarithmic visual chart. Speed was defined as the change in viewing angle per second. In each trial, the letter E traversed the screen from the midpoint of the left edge toward the right before disappearing.

All tests were conducted binocularly with participants wearing their habitual spectacles at a fixed viewing distance of 1 meter. Four DVA test sessions were performed sequentially at motion speeds of 20, 40, 60, and 80 degrees per second (dps). These speeds were selected based on our prior research to account for variations in eye movement strategies (Wang et al., 2022). The presentation time of the target at 20, 40, 60, and 80 was about 1.6 s, 0.8 s, 0.5 s, and 0.4 s. A head and chin rest stabilized the participant’s head during testing. Participants verbally reported the opening direction of the optotype letter E-up, down, left, or right-in a four-alternative forced-choice task. Pretraining trials began with optotypes three steps larger than the static visual acuity to familiarize participants with the procedure and motion pattern. In the formal test, eight optotypes of the same size but random opening directions were presented sequentially with 2-s intervals. The size was reduced by one step if five or more optotypes were correctly identified, continuing until fewer than five were correct. DVA was calculated using the following formula and presented in logMAR (Wang et al., 2022). A is the smallest optotype size (in logMAR) at which at least five of eight answers were correct, while b represents the number of correct responses at the next smaller size.


$$\mathrm{DVA}=A+\frac{0.1}{8}*b$$

### 2.3 Eye movement recording and processing

During the DVA test, we recorded binocular eye position using a desk-top-mounted eye tracker (Tobii Pro Fusion 120, Tobii Pro AB, Sweden) at a 120 Hz sampling rate. The accuracy of the eye tracker was 0.4 degree and the systemic delay was less than 3 frames. Eye movements were analyzed offline, with the positional data smoothed over time via the convolve function from Python’s scipy package. Velocity and acceleration were derived by differentiating the eye position data. The resulting traces generally showed an initial fixation phase, followed by alternating saccades and smooth pursuit. Saccades were first identified and excluded when three consecutive samples surpassed a velocity threshold of 50 dps, with saccade onset and offset determined as the nearest zero-crossing points in the acceleration trace, respectively (Fooken et al., 2022). The remaining trajectory was further segmented into initial fixation and several smooth pursuit segments using piecewise linear regression, where smooth pursuit was classified as eye movement in a consistent direction. The focus of the segmented regression line is used to determine the end of fixation and the onset of the first smooth pursuit. The fixation segment was the trace from the optotype onset to the first smooth pursuit or saccade. Among the smooth pursuit segments, the longest segment was extracted for parameter analysis. The region of interest (ROI) was defined as the square area enclosing the optotype. We applied manual corrections to rectify minor detection inaccuracies.

For each trial in the formal test, we computed the following eye movement parameters: (1) Position error (degree) represents the mean absolute horizontal deviation between eye position and target center across the entire movement phase. (2) ROI time (s) is the cumulative duration the eye position remained inside the ROI. (3) ROI surpassing time (s) indicates the total time the eye position exceeded the ROI boundary. (4) Saccade episode (number) counts the total saccadic events during the trial. (5) Saccade duration (s) sums the time of all saccadic segments. (6) Saccade displacement (degree) measures the total angular distance covered by saccades. (7) Displacement per saccade (degree) gives the mean angular distance per saccadic segment. (8) Pursuit duration (s) totals the time of smooth pursuit segments. (9) Pursuit position error (degree) denotes the mean absolute horizontal deviation during smooth pursuit segments. (10) Max velocity in open-loop (dps) identifies the peak velocity within the initial 140 ms of smooth pursuit (Fooken et al., 2016). (11) Velocity in closed-loop (dps) is the average velocity between 200 and 400 ms after pursuit onset. Trials exhibiting invalid eye tracking data, signal loss, or interruptions were excluded. Smooth pursuit-related parameters were omitted when pursuit was not detected in the gaze trace, which occurred primarily during the DVA test at 80 dps.

### 2.4 Statistical analysis

Data analysis was conducted using IBM SPSS Statistics (version 23.0; IBM Corp., USA). Continuous variables are presented as mean ± standard deviation (SD), while categorical variables are expressed as counts and percentages. Normality was assessed using the Shapiro–Wilk test. A linear mixed model was used to compare the eye movement parameters during the DVA test at different velocities. The velocity was set as the repeated factor, and the participant was treated as the subject to include a random intercept. The association between DVA and eye movement parameters was examined using Pearson or Spearman’s correlation, depending on data normality.

A generalized linear mixed model analyzed the influence of eye movement parameters on the correctness of identifying optotype opening direction. The dependent variable was identification correctness per trial, a binary variable with correct judgment as the reference. The primary subject was the participant, and the secondary subject was optotype size, accounting for repeated measurements under the same participant and size. The model incorporated random intercepts for both primary and secondary subjects. Post-hoc analysis employed Bonferroni correction for multiple comparisons across the four target velocities, with a *p*-value < 0.05 considered statistically significant.

## 3 Results

### 3.1 Demographic data and DVA

The study comprised 20 participants with a mean age of 22.8 ± 3.4 years (range: 20–33), 47% of whom were male. The mean spherical refraction was −4.85 ± 2.10 diopters (D), and the mean cylinder power was −1.07 ± 0.67 D. Mean corrected distance visual acuity (CDVA) with habitual spectacles measured 0.05 ± 0.12 LogMAR.

DVA at 20 dps was 0.13 ± 0.11 LogMAR, significantly better than the values recorded at 40 dps (0.25 ± 0.11 LogMAR, *p* < 0.001), 60 dps (0.29 ± 0.10 LogMAR, *p* < 0.001), and 80 dps (0.28 ± 0.12 LogMAR, p < 0.001). No significant differences in DVA were observed among the 40, 60, and 80 dps conditions (*p* > 0.05 for all comparisons).

### 3.2 Eye movements during the DVA test at different speeds

**Figure 1:**
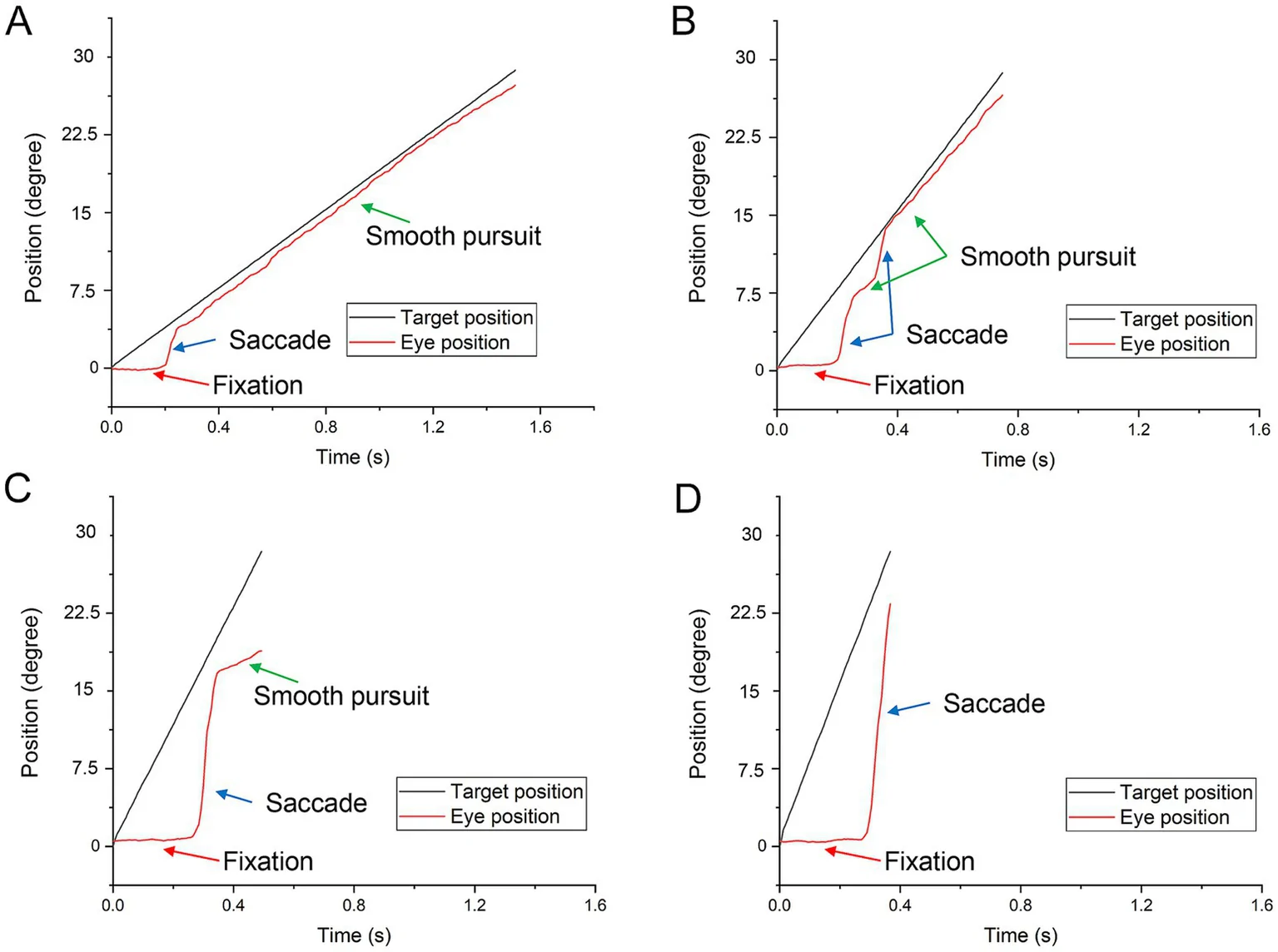
Typical gaze traces during DVA tests of different motion speeds. **(A)** 20 dps; **(B)** 40 dps; **(C)** 60 dps; **(D)** 80 dps. Dps, degrees per second.

Figure 1 illustrates typical gaze traces recorded during DVA tests across different target speeds. At 20 dps, eye movements typically began with an initial fixation, followed by a rapid saccade, and transitioned into maintenance with a long, smooth pursuit to the end of target movement. Under the 40-dps condition, gaze behavior started with a fixation, followed by a repeated alternating saccade to acquire the target and smooth pursuit. At 60 dps, the gaze pattern resembled that at 40 dps, although the repeated cycle was fewer and the smooth pursuit phase was shorter. When the target moved at 80 dps, the predominant pattern consisted of an initial fixation and a saccade, with the gaze trace terminating either before smooth pursuit could begin or after only a brief period of pursuit.

Table 1 summarizes the eye movement parameters recorded during the DVA test at different velocities and the line graphs are presented in Supplementary Figure S1. The positional error increased significantly with speed, rising from 1.08 ± 0.31 degree at 20 dps to 9.60 ± 2.09 degree at 80 dps (*p* < 0.05 for pairwise comparisons). At 20 dps, the duration for which eye position remained within the ROI and exceeded it was longer than at all other tested velocities (*p* < 0.001 in each case).

**Table 1:** The eye movement parameters during the DVA test at different velocities.

Eye movement parameters	20 dps	40 dps	60 dps	80 dps	*p-*value
Position error (degree)	1.08 ± 0.31	2.39 ± 0.47	5.96 ± 2.18	9.60 ± 2.09	<0.001
ROI duration (s)	0.039 ± 0.037	0.006 ± 0.003	0.004 ± 0.002	0.002 ± 0.001	<0.001
ROI surpassing duration (s)	0.14 ± 0.11	0.07 ± 0.05	0.05 ± 0.04	0.04 ± 0.05	<0.001
Saccade					
Saccade episode (number)	1.72 ± 0.39	2.99 ± 0.52	1.91 ± 0.76	0.94 ± 0.54	<0.001
Saccade duration (s)	0.03 ± 0.01	0.10 ± 0.02	0.10 ± 0.04	0.04 ± 0.03	<0.001
Saccade displacement (degree)	2.91 ± 1.48	9.96 ± 2.85	14.57 ± 6.53	8.74 ± 5.51	<0.001
Displacement per saccade (degree)	1.65 ± 0.93	3.46 ± 1.02	8.36 ± 3.35	9.74 ± 4.72	<0.001
Smooth pursuit					
Pursuit duration (s)	1.25 ± 0.03	0.46 ± 0.04	0.17 ± 0.07	0.06 ± 0.04	<0.001
Pursuit position error (degree)	0.89 ± 0.30	1.90 ± 0.40	4.72 ± 2.02	8.61 ± 3.21	<0.001
Max velocity in open-loop (dps)	41.73 ± 2.46	50.58 ± 2.73	42.44 ± 9.97	34.96 ± 9.70	<0.001
Mean velocity in closed-loop (dps)	19.44 ± 1.05	30.66 ± 5.20	21.73 ± 6.61	7.42 ± 3.18	<0.001

Dps, degrees per second; DVA, dynamic visual acuity; ROI, regions of interest.

Regarding saccade parameters, saccade episode, duration, and displacement all followed an inverted-*U* pattern, with their peaks occurring at 40–60 dps. The number of saccade episodes reached its maximum at 40 dps (*p* < 0.05 for pairwise comparisons), while saccade displacement was greatest at 60 dps compared with other velocities (*p* < 0.05 for pairwise comparisons). Saccade duration was significantly longer at 40 and 60 dps than at 20 and 80 dps (*p* < 0.05, respectively). In contrast, displacement per saccade increased steadily with speed (*p* < 0.001). Effective smooth pursuit duration declined as speed increased (*p* < 0.001), whereas pursuit position error rose (*p* < 0.001). Maximum velocity in the open-loop phase and mean velocity in the closed-loop phase both exhibited an inverted-*U* pattern, peaking at 40 dps (*p* < 0.05 for all analyses).

### 3.3 Correlation between eye movement parameters and DVA

Table 2 presents the correlations between eye movement parameters and DVA across different velocities. At 20 dps, DVA (logMAR) exhibited a negative correlation with saccade displacement (*r* = −0.523, *p* = 0.022) and displacement per saccade (*r* = −0.528, *p* = 0.020). No significant associations between any parameters and DVA were observed at 40 (*p* > 0.05 for each). At 60 dps, DVA was positively correlated with saccade episode (*r* = −0.628, *p* = 0.0004), and negatively correlated with displacement per saccade (*r* = −0.565, *p* = 0.012). At 80 dps, DVA (logMAR) was positively associated with saccade episode (*r* = 0.521, *p* = 0.022), saccade duration (*r* = 0.500, *p* = 0.029), and saccade displacement (*r* = 0.483, *p* = 0.036). Furthermore, DVA (logMAR) at this velocity also showed significant positive associations with pursuit duration (*r* = 0.452, *p* = 0.026) and max velocity in open-loop (*r* = 0.470, *p* = 0.035).

**Table 2:** The correlation analysis between DVA and eye movement parameters.

Eye movement parameters		20 dps	40 dps	60 dps	80 dps
Mean position error	*r*	−0.329	−0.238	−0.475	−0.099
	*p*	0.170	0.327	0.060	0.686
ROI duration	*r*	0.308	0.357	0.198	−0.011
	*p*	0.199	0.134	0.416	0.963
ROI surpassing duration	*r*	0.120	0.137	0.123	−0.007
	*p*	0.623	0.575	0.615	0.977
Saccade					
Saccade episode	*r*	0.036	0.176	0.628	0.521
	*p*	0.884	0.472	0.004	0.022
Saccade duration	*r*	−0.320	0.240	0.256	0.500
	*p*	0.182	0.322	0.291	0.029
Saccade displacement	*r*	−0.523	0.024	0.033	0.483
	*p*	0.022	0.922	0.892	0.036
Displacement per saccade	*r*	−0.528	−0.095	−0.565	0.188
	*p*	0.020	0.699	0.012	0.440
Smooth pursuit					
Pursuit duration	*r*	0.256	0.233	0.264	0.452
	*p*	0.291	0.336	0.274	0.026
Pursue position error	*r*	−0.051	−0.154	−0.306	−0.254
	*p*	0.836	0.528	0.202	0.324
Max velocity in open-loop	*r*	0.033	0.024	0.192	0.450
	*p*	0.892	0.922	0.431	0.035
Mean velocity in closed-loop	*r*	0.149	−0.235	−0.036	−0.522
	*p*	0.544	0.364	0.932	0.288

Dps, degrees per second; DVA, dynamic visual acuity; ROI, regions of interest.

### 3.4 Associations between eye movement parameters and optotype identification accuracy

Generalized linear mixed models evaluated the influence of eye movement parameters on individual optotype identification accuracy, with the results presented in Table 3. At 20 dps, increased saccade episode (*β* = 0.540, *p* < 0.001) and duration (*β* = 27.333, p < 0.001) were associated with a higher likelihood of identification error. At 80 dps, higher velocity during the open-loop phase was associated with improved identification error odds (*β* = 0.030, *p* = 0.016).

**Table 3:** The generalized mixed linear model for optotype identification correctness^*^.

Eye movement parameters		20 dps	40 dps	60 dps	80 dps
Mean position error	Coefficient	0.047	−0.176	−0.041	−0.086
	*p*	0.241	0.246	0.407	0.052
ROI duration	Coefficient	4.406	−21.34	−8.595	38.002
	*p*	0.147	0.095	0.704	0.173
ROI surpassing duration	Coefficient	1.185	−1.721	−4.234	−2.005
	*p*	0.114	0.276	0.060	0.379
Saccade					
Saccade episode	Coefficient	0.540	−0.086	−0.157	0.227
	*p*	<0.001	0.414	0.199	0.131
Saccade duration	Coefficient	27.333	−1.998	1.173	3.265
	*p*	<0.001	0.600	0.653	0.314
Saccade displacement	Coefficient	−0.001	−0.026	0.011	0.011
	*p*	0.954	0.339	0.419	0.479
Displacement per saccade	Coefficient	0.175	−0.005	0.027	−0.013
	P	0.135	0.932	0.218	0.567
Smooth pursuit					
Pursue duration	Coefficient	−1.796	0.738	−1.053	0.167
	*p*	0.221	0.578	0.369	0.904
Pursue position error	Coefficient	0.812	−0.168	−0.030	−0.037
	*p*	0.128	0.314	0.500	0.412
Max velocity in open-loop	Coefficient	−0.001	0.010	−0.003	0.030
	*p*	0.932	0.458	0.712	0.016
Mean velocity in closed-loop	Coefficient	−0.076	0.016	0.092	0.818
	*p*	0.231	0.620	0.056	0.194

^*^The dependent variable was the identification correctness for each trial (a binary variable), and the correct judgment was set as the reference.

Dps, degrees per second; DVA, dynamic visual acuity; ROI, regions of interest.

## 4 Discussion

DVA serves as a valuable indicator of functional vision in clinical ophthalmology and has been applied in patients with refractive error, cataract, and dry eye (Wang et al., 2022, 2023b). Eye movements are essential for localizing targets and stabilizing images on the retina (Babu et al., 2005). Yet their patterns remain poorly characterized during clinical DVA testing. This study characterized eye movement patterns with an eye-tracker during DVA tests at different velocities and examined the associations between eye movement parameters and perceptual outcomes. We observed that eye movement patterns varied systematically with velocity, reflecting a speed-dependent trade-off between smooth pursuit and saccades. Eye movement patterns, particularly saccadic efficiency, influence dynamic visual performance in a speed-dependent manner.

DVA is significantly influenced by target speed and decreases as velocity increases (Wang et al., 2024). In this study, DVA was highest at 20 dps compared to other velocities. This finding aligns with previous reports (Wang et al., 2022, 2024). The variation in dynamic vision performance likely stems from differences in eye movement patterns across speeds. Gaze traces revealed that position error increased significantly while smooth pursuit duration decreased with higher target speeds. These observations support the view that stable smooth pursuit maintains the target image near the fovea, thereby reducing retinal slip and motion blur to improve acuity (Spering and Montagnini, 2011; Moulder et al., 2013; Uchida et al., 2012). At higher speeds, the eyes fail to maintain precise alignment, leading to increased retinal slip and degraded DVA. When smooth pursuit becomes insufficient, catch-up saccades are triggered to correct position error (de Brouwer et al., 2002; Palidis et al., 2017). Trajectory analysis further indicated that both saccade duration and displacement increase substantially during high-speed tracking.

Significant variations in gaze traces were observed during DVA tests conducted at different motion speeds. The present research used continuous motion starting from the screen edge without a step-ramp design, and manifested several separate segments of smooth pursuit in the gaze trace. The analyzed smooth pursuit represents the longest segment, but not necessarily the first pursuit trace. During the 20-dps condition, smooth pursuit could be maintained following the initial saccade throughout the target’s movement. The observed gaze pattern matches earlier reports where participants successfully tracked targets using smooth pursuit eye movements at 20 dps (de Brouwer et al., 2002). At higher speeds, maintaining long-lasting smooth pursuit becomes more challenging, leading to reduced tracking accuracy. When target velocity increased to 40 dps, we noted periodic alternations between smooth pursuit and saccadic movements to correct positional errors. As velocities reached 60 and 80 dps, pursuit durations further shortened, and saccades became the primary mechanism for error correction, revealing the inherent speed limitations of smooth pursuit in human vision. The decline in pursuit performance at higher speeds may reflect both the increasing velocity demands and the progressively shorter presentation times available for pursuit initiation and maintenance. Meyer et al. (1985) demonstrated that human smooth pursuit velocity can reach 100 degrees per second under optimal conditions, while van den Berg and Collewijn (1986) showed that the pursuit accuracy degrades when trajectory constraints limit predictive control. In our paradigm, the brief presentation time at 60 and 80 dps may not allow sufficient time for pursuit to reach steady-state, contributing to the observed performance decline. In reviewing individual gaze traces, the eye movement pattern showed generally consistent trends across participants, particularly the shift from smooth pursuit dominance at 20 dps to saccade prominence at 80 dps. However, some individual variability was observed in the exact number and timing of catch-up saccades, which is further reflected in the standard deviations of the eye movement parameters. This variability may be clinically relevant, as individual differences in oculomotor efficiency could influence DVA outcomes in patient populations.

Because the optotype appeared at predictable fixed intervals and moved in a constant direction and velocity within each speed condition, anticipatory eye movements may have been involved. Participants could have used temporal and spatial expectations to initiate predictive smooth pursuit or saccades before target onset, potentially reducing the reactive component of tracking. This predictive strategy could influence DVA outcomes by reducing initial position error, but could also introduce systematic biases if the anticipated timing or trajectory does not perfectly match the actual stimulus. Future studies using randomized inter-trial intervals or variable start positions could help disentangle reactive from anticipatory components of eye movements in clinical DVA testing. To more directly examine the effect of presentation period on acuity independent of target motion, future studies could present stationary optotypes for durations matched to the presentation periods used here. Such a control condition would help separate the contribution of motion-induced eye movement demands from that of simple temporal sampling constraints on acuity performance.

Eye movement patterns significantly influence dynamic visual performance. At a target speed of 20 dps, this study found that larger saccade displacements and greater displacement per saccade correlated with better DVA. Conversely, a higher number of saccade episodes and increased saccade duration raised the likelihood of optotype identification errors. These results suggest that the initial saccade, executed before smooth pursuit to correct positional error, is critical for identifying moving optotypes at low speeds. Remarkable dynamic vision at low speeds thus depends on timely and precise initial saccades rather than prolonged or repeated ones. For 60 dps test, greater saccade episode and short displacement per saccade were correlated with poor DVA. For DVA at a high speed of 80 dps, however, greater saccade episode count, duration, and displacement were associated with poorer performance. Longer pursuit duration and higher open-loop velocity also correlated with reduced DVA and increased identification errors at this speed. Palidis et al. (2017) similarly reported that observers with greater overall saccade distances and higher velocity gain during smooth pursuit exhibited worse dynamic vision at high target speeds. Though we did not categorize reverse saccades in the analysis, the result suggests that the pattern of saccadic activity may be more detrimental than the absolute positional deviation. The repeated or prolonged saccadic activity interrupts foveal image stabilization and reduces the time available for visual sampling, independent of the average position error magnitude. Therefore, an optimized saccade strategy is essential for achieving high dynamic vision performance, characterized by rapid and accurate saccadic movements that minimize positional error.

Notably, Unlike Palidis et al. (2017), a direct correlation between position error and DVA was not observed. The discrepancy may also stem from technical and paradigm differences. The low sampling rate in the present research may reduce sensitivity to transient position errors, compared to 1,000 Hz sampling in previous research. The brief target presentation at high speed may have precluded sufficient tracking time for the position error to stabilize as a meaningful predictor. The continuous-motion paradigm without step-ramp onset may also have introduced greater position variability. Additionally, the 120 Hz sampling rate of our eye tracker limits detection of small saccades, which may be particularly relevant during maintenance of smooth pursuit. The limitation is that some tiny catch-up saccades may go undetected, potentially affecting the accuracy of pursuit parameter estimation and saccade episode counts. Future studies with higher-resolution eye tracking and extended presentation times under clinical test settings could clarify the relationship. Our findings applied specifically to dynamic-object DVA with head fixation. Palidis et al. (2017) found no correlation between dynamic-object and static-object DVA, indicating these tests assess different capacities. Whether eye movement strategies in static-object DVA show similar speed-dependent variation remains to be determined.

This study has several limitations. First, the relatively small sample size may limit the statistical power for detecting subtle associations and generalizing the findings, given the inherent variability in eye movement patterns across individuals. Second, the participant cohort was restricted to young adults with normal or corrected-to-normal visual acuity. Future studies should therefore include a broader demographic, enrolling individuals of different ages and patients with ocular conditions that affect DVA and eye movements. Third, eye movement duration varied across tests performed at different velocities. During high-velocity DVA testing, the visual target’s presentation time was sometimes too short, allowing the target to complete its trajectory before eye movement initiation. Fourth, the low sampling rate of the eye-tracker limits our ability to detect small saccades and accurately characterize brief pursuit segments, particularly at higher velocities where catch-up saccades may be more frequent but smaller in amplitude. The technical constraint may lead to incomplete characterization of the saccade-pursuit interaction. Fifth, the current DVA test assessed only horizontal motion; future work could explore vertical or diagonal motion paradigms. Sixth, we only analyze the character of saccades (episode, duration, and displacement), but do not categorize the type of saccades. Future studies could examine reverse saccade strategies in clinical DVA testing.

This study characterized eye movement patterns across target velocities used in the clinical DVA test, revealing that strategies varied systematically with speed. At 20 dps, stable smooth pursuit following saccades predominated, supporting optimal DVA performance. As target speed increased, smooth pursuit duration shortened and saccades became dominant, coinciding with a decline in DVA. At low speeds, rapid and efficient saccades supported improved dynamic visual performance. In contrast, high amplitude saccades and high pursuit velocity disrupted the tracking and impaired the performance. These findings underscore the relevance of eye movement behavior in DVA assessment and help establish a foundation for refining standardized testing protocols and their clinical applications.

## Data Availability

The raw data supporting the conclusions of this article will be made available by the authors, without undue reservation.
