# Supplementary material for: Efficient saccade enhances performance in clinical dynamic visual acuity test
Source: Front Neurosci. 2026 Aug 7;20:1834160. doi: 10.3389/fnins.2026.1834160 (PMC13493566; doi:10.3389/fnins.2026.1834160)
Supplement: Supplementary file 1 [file Supplementary_file_1.docx]

Figure S1. The eye movement parameters during the DVA test at different velocities. A. Position error; B. ROI duration; C. ROI surpassing duration; D. Saccade episode; E. Saccade duration; F. Saccade displacement; G. Displacement per saccade; H. Pursuit duration; I. Pruisuit position error; J. Max velocity in open-loop; K. Mean velocity in closed-loop. Dps, degrees per second; DVA, dynamic visual acuity; ROI, regions of interest.
